# Fine Tuning of the Mechanical Properties of Bio-Based PHB/Nanofibrillated Cellulose Biocomposites to Prevent Implant Failure Due to the Bone/Implant Stress Shielding Effect

**DOI:** 10.3390/polym15061438

**Published:** 2023-03-14

**Authors:** Martina Ferri, Emanoele Maria Santos Chiromito, Antonio Jose Felix de Carvalho, Davide Morselli, Micaela Degli Esposti, Paola Fabbri

**Affiliations:** 1 Department of Civil, Chemical, Environmental and Materials Engineering, Università di Bologna, Via Terracini 28, 40131 Bologna, Italy; 2 National Interuniversity Consortium of Materials Science and Technology (INSTM), Via Giusti 9, 50121 Firenze, Italy; 3 Department of Materials Engineering, Engineering School of São Carlos, University of São Paulo, Av. João Dagnone, 1100, São Carlos 13563-120, SP, Brazil

**Keywords:** poly(hydroxyalkanoate)s, poly(ethylene glycol), NFC, bone regeneration, tissue engineering, mechanical properties mismatch

## Abstract

A significant mechanical properties mismatch between natural bone and the material forming the orthopedic implant device can lead to its failure due to the inhomogeneous loads distribution, resulting in less dense and more fragile bone tissue (known as the stress shielding effect). The addition of nanofibrillated cellulose (NFC) to biocompatible and bioresorbable poly(3-hydroxybutyrate) (PHB) is proposed in order to tailor the PHB mechanical properties to different bone types. Specifically, the proposed approach offers an effective strategy to develop a supporting material, suitable for bone tissue regeneration, where stiffness, mechanical strength, hardness, and impact resistance can be tuned. The desired homogeneous blend formation and fine-tuning of PHB mechanical properties have been achieved thanks to the specific design and synthesis of a PHB/PEG diblock copolymer that is able to compatibilize the two compounds. Moreover, the typical high hydrophobicity of PHB is significantly reduced when NFC is added in presence of the developed diblock copolymer, thus creating a potential cue for supporting bone tissue growth. Hence, the presented outcomes contribute to the medical community development by translating the research results into clinical practice for designing bio-based materials for prosthetic devices.

## 1. Introduction

Life expectancy, which is the key metric for assessing population health, has increased rapidly in recent decades. At the same time, increasing age can be considered one of the strongest predictors of the development and progression of bone diseases. Several organizations warn of the importance of prioritizing public policies focused on osteoarticular problems and the need to develop new materials and improve the quality of people’s lives [1,2,3,4].

At present, the most used materials for prostheses fabrication in the orthopedic field are metals, in particular stainless steels, titanium and its alloys, and cobalt–chromium–molybdenum alloys. These materials show high biocompatibility and resistance to corrosion. Moreover, they have an excellent mechanical performance as well as high resistance to fracture and fatigue, which make them widely used as structural components aiming at the replacement, reinforcement, or stabilization of bones that have to bear both high tensile and compression loads [5,6,7]. However, metal prostheses are associated with several complications, such as implant loosening, infections, and the stress shielding effect [8]. The latter phenomenon is the direct consequence of the different mechanical properties between the bone tissue and implanted material. Specifically, it is well known that healthy bone remodels in response to the loads it is placed under. When stiffer artificial metallic implants are used instead of native bone, a significant alteration of the load distribution occurs. This phenomenon affects the appropriate bone remodeling process, inducing the formation of a weaker bone in the area that is no longer physiologically loaded (stress-shielded zone) and of denser bone in the region bearing stresses greater than physiological. Therefore, the careful tailoring of the mechanical properties, with regard to the specific load required by a certain bone, is of primary importance in order to reduce the mismatch between the two materials.

Emerging biomaterials for bone replacement and regeneration are entering the orthopedic field thanks to their set of physical, chemical, and biological properties that allow them to achieve the appropriate function, in addition to actively stimulating the desired tissue response, thus making the recovery more rapid and efficient [6,8]. Among them, biocompatible and bioresorbable polymer-based materials [9,10] can be easily 3D-manufactured in order to fabricate a patient-specific replacement of the injured area. Furthermore, polymers show easily tunable mechanical properties and, thanks to their bioresorbability, a second surgery can be avoided since the implanted material is resorbed by the body [9].

Among the biobased and bioresorbable polyesters proposed for biomedical applications, poly(3-hydroxybutyrate) (PHB) is one of the most studied polymers [11,12,13,14], thanks to its well-known biocompatibility, piezoelectric properties, and ability to support bone tissue growth [15,16,17].

Compounding with suitable additives represents a valuable strategy to modulate its final properties, and biobased substances are preferred in order to preserve its renewability. The high abundance and renewable origin of cellulose have made nano-fibrillated cellulose (NFC) [18] particularly attractive. Moreover, thanks to NFC’s high specific surface area, polymer/NFC interactions are enhanced, leading to improved mechanical properties [19,20]. Furthermore, it is worth noting that the addition of hydrophilic NFC represents an effective hydrophilization approach for improving the water affinity of PHB [21], which contributes to the definition of a favorable environmental cue that supports cell attachment and proliferation.

In addition, compounding PHB with suitable plasticizers [22] or polymers, such as poly(ethylene glycol) (PEG), can decrease the well-known high crystallinity of PHB and consequent stiffness, which limits its wide applicability [23]. Alongside increasing its flexibility and facilitating its processing, the hydrophilicity of PEG can be exploited to disperse the NFC into the hydrophobic PHB.

Herein, we propose the design and synthesis of a PHB–PEG diblock copolymer able to enhance the compatibility between the components of the blend. The copolymer was obtained through a solvent-free process that did not require a complicated workup. The copolymer addition allows us to obtain homogeneous blends with well-distributed NFC and the desired fine-tuning of the mechanical properties. Two formulations are herein proposed: (i) a PHB–PEG blend reinforced with NFC and (ii) the addition of the developed PHB–PEG diblock copolymer to improve the compatibility between the NFC in the PHB. The obtained blends are fully characterized from a mechanical point of view and the wettability was tested in order to evaluate the potential application of these materials to tailor their final properties and reduce the stress shielding effect.

## 2. Experimental

### 2.1. Materials

PHB was purchased from Sigma Aldrich (custom synthesis, M_n_ 71,600, M_w_ 245,100, Milan, Italy) and carefully purified in order to remove impurities before the preparation of the copolymer (detailed procedure described elsewhere [10]). PEG 4000 (PEG, synthesis grade) was purchased from Scharlau and PEG 1900 monomethyl ether (mPEG) was purchased from Alfa Aesar (Kandel, Germany), and both were used as received. Nanofibrillated cellulose (NFC) in aqueous suspension, obtained from eucalyptus bleached kraft wood pulp (dry matter content: 3.2 wt.%, negligible lignin content, hemicellulose content: 10–15%, fiber diameters of 10–50 nm and a few micrometers in length), was supplied by Suzano Papel e Celulose S.A. (Sao Paulo, Brazil) [24,25]. Chloroform (CHCl_3_, HPLC grade), methanol (MeOH, ≥99.8%), titanium (IV) butoxide (TBT, 97%), toluene (HPLC grade), and celite (Standard Super Cel^®^ fine) were purchased from Sigma-Aldrich (Milan, Italy) and used as received without further purification. Representative photographs of as-received PHB, purified PHB, and mPEG are shown in Figure S1.

### 2.2. Preparation of NFC

An aqueous suspension of NFC cannot be directly mixed with PHB due to the very low solubility of the polymer in water. On the other hand, the NFC aqueous suspension cannot be dried and redispersed since NFC typically creates large aggregates that cannot then be homogeneously dispersed in PHB. To overcome this problem, we proposed an innovative approach that limits the NFC aggregation that typically occurs during the drying step. In detail, 62.5 g of an aqueous suspension of NFC (3.2 wt.%) was added to 80 g of PEG 4000 solution (10 wt.% in distilled water). The suspension was kept under magnetic stirring for 15 min and further homogenized by an ULTRA-TURRAX^®^ stirrer (IKA, Staufen, Germany) operating at 8500 rpm, for 20 min. The aqueous PEG/NFC suspension was freeze-dried by an Epsilon 2–4 LSCplus (Martin Christ, Osterode am Harz, Germany) in order to remove water. The prepared PEG/NFC (80/20, wt.%/wt.%, 10 g) cake was then cryo-grinded (using liquid nitrogen) to obtain a fine powder, which could then be used as a filler in an extrusion system.

### 2.3. PHB–Block–mPEG Copolymer Synthesis

The procedure for the copolymer (PHB–block–mPEG copolymer, COP) preparation was conducted by adjusting the previously proposed synthesis reported by Ravenelle and Marchessault [26]. In brief, 10 g of purified PHB and 10 g of mPEG 1900 were loaded in a Schlenk reactor under magnetic stirring. When the vacuum was created, the reactor was plunged into a preheated oil bath at 190 °C until the melt viscosity of the polymeric mixture was appropriate to create homogeneous stirring. The mixture was stirred for 10 min and nitrogen was flowed in the reactor. Then, 0.01 g of TBT (0.1 wt.% with respect to PHB amount) was dissolved in 100 μL of toluene and added to the reaction mixture, according to the previously reported study [27]. Fifteen minutes after the TBT addition, the reaction was rapidly quenched in an ice bath. The reaction mixture was then added to Milli-Q water and stirred overnight at room temperature. The obtained pale-yellow solid copolymer (photograph in Figure S1) was collected by filtration. The dried solid was weighed to calculate the yield that was in the range of 94–98 wt.%. The yield was calculated as the weight of the product obtained versus the initial weights of PHB and PEG.

### 2.4. Samples Preparation

The PHB/PEG blend was obtained by the direct mixing of the components by melt processing. NFC-containing composites were prepared by blending the freeze-dried PEG/NFC with PHB (two proportions: 0.5 and 1 wt.% NFC) by extrusion using a co-rotating twin-screw extruder (Icma San Giorgio, San Giorgio su Legnano, Italy, 25 mm screw diameter). A second series of compounds was prepared using the same procedure described above; however, 5 wt.% of copolymer COP with respect to the PHB was added to improve the compatibility between the two polymers PHB and PEG. All details about the prepared samples and their formulations are reported in Table 1. After the processing stage, dog-bone samples (representative photographs presented in Figure S2) were prepared by injection molding.

### 2.5. Characterization

Sample morphology and NFC dispersion were investigated by a scanning electron microscope Nova NanoSEM 450 (FEI) equipped with a field-emission gun (FESEM). The cryo-fractured (by liquid nitrogen) cross-sections of the composites were analyzed. PEG/NFC particles were also examined by fixing them in a conductive carbon tape and all samples were sputter-coated with 10 nm of gold. FESEM images were analyzed by Fiji/ImageJ2 (version 2.3.0) open-source software to determine the mean particle sizes and related standard deviations (approx. 30 particles per sample were analyzed).

Infrared spectroscopy (FT-IR) investigations were performed by a PerkinElmer Spectrum Two Spectrometer equipped with a diamond crystal in attenuated total reflectance (ATR) mode. Measurements were performed in the range of 4000 to 400 cm^−1^ at room temperature; the spectral resolution was 4 cm^−1^ and the number of scans was 64 for each spectrum. Spectra were processed with Spectrum 10 software (PerkinElmer, Milan, Italy).

Proton Nuclear Magnetic Resonance measurements (^1^H-NMR) were conducted at room temperature on Varian Unity 400 operating at 400 MHz; 28 scans were recorded with a relaxation delay time (T_1_) of 1 s. All samples were dissolved in CDCl_3_ (approx. 10 mg in 0.85 mL). The peak at 7.26 ppm associated with CDCl_3_ was used as the reference. All spectra were processed with VnmrJ software (Varian, Inc.). The percentages of PHB and PEG blocks of the copolymer were calculated according to the previously reported approach by Ravenelle and Marchessault [26]. All calculations were reported in the Supplementary Materials.

The molecular weight of the synthesized copolymer was determined by gel-permeation chromatography (GPC) with an Agilent 1260 Infinity instrument (Agilent Technologies, Cernusco sul Naviglio, Italy) G1322A 1260 Degasser, G1310B 1260 Isocratic Pump, G1316A 1260 TCC Thermostated Column Compartment, G1362A 126 RID Reflective Index Detector, G1328C 126 Manual Injector); the RID and column compartment were thermostatically controlled at 35 °C ± 0.2 °C. The instrument was equipped with a PLgel MiniMIX-A column (20 μm particle size, 4.6 × 250 mm) coupled with a Tosoh TSKgel SuperMultipore HZ- M column (4 μm particle size, 4.6 × 150 mm); the columns were preceded by a low-dispersion in-line filter (frit porosity: 0.2 μm). CHCl_3_ was used as the mobile phase at a flow rate of 0.2 mL·min^−1^ and toluene was used as the internal standard (0.1 μL·mL^−1^), with a run time of 37 min. The data were processed with Agilent GPC/SEC software, version A.02.01, using a calibration curve obtained with monodispersed polystyrene standards (EasiCal PS-1 Agilent kit).

Before starting the mechanical and thermal properties’ characterization, all specimens were conditioned at 45 °C for 72 h in an oven. This was necessary so that all samples had comparable conditions and the effects were minimized due to well-known secondary crystallization that typically occurs after manufacturing in PHB-based materials.

The tensile strength and modulus of the neat polymer, blends, and composites were analyzed in order to evaluate the effects of different compositions. The tests were performed on a 5966 Universal Testing System (Instron) equipped with a 10 kN load cell at a deformation rate of 1 mm·min^−1^ and 50 mm extensometer. The measurements of the samples were in accordance with the standard methodology ASTM D 638.

Flexural tests were conducted in an Instron 5966 Universal Material Testing Machine equipped with a 5 kN load cell at a deformation rate of 2 mm·min^−1^ and 64 mm distance between the specimen supports. The measurements of the samples were in accordance with the standard methodology ASTM D 790.

Charpy impact strength tests were conducted on a Ceast Resil Impactor (Instron, Norwood, MA, USA) using a hammer mass that had impact with an energy of 5 J on notched specimen bars, according to the ASTM D 6110-10 standard. Measurements were performed at 23 °C and a 50% humidity rate.

Hardness was measured with a Shore D hardness test (Affri, Induno Olona, Italy) under a load of 5 kg and a retention time of 15 s, according to the standard methodology ASTM D 2240. The hardness was determined based on the mean value of six measures for each sample.

Dynamic mechanical behavior was investigated by Dynamic Mechanical Analysis (DMA) on a Q800 analyzer (TA Instruments, Sesto San Giovanni, Italy) in single cantilever mode. Specimens (40 mm × 15 mm × 4 mm) were analyzed from −60 to +60 °C with a heating rate of 3 °C·min^−1^ and oscillation frequency of 1 Hz.

The static water contact angle (WCA) was investigated to explore the wetting behavior of the samples. The test was conducted by a Krüss DSA 30 contact angle goniometer (Nürnberg, Germany) equipped with a digital camera. In detail, 2 μL of Milli-Q water droplets were deposited on the surface of the samples and one frame was recorded. No evidence of water absorption was observed for any of the tested materials and within the measurement time. The average WCA values and related standard deviations were calculated for each sample based on at least 8 measurements.

## 3. Results and Discussion

**Figure 1:**
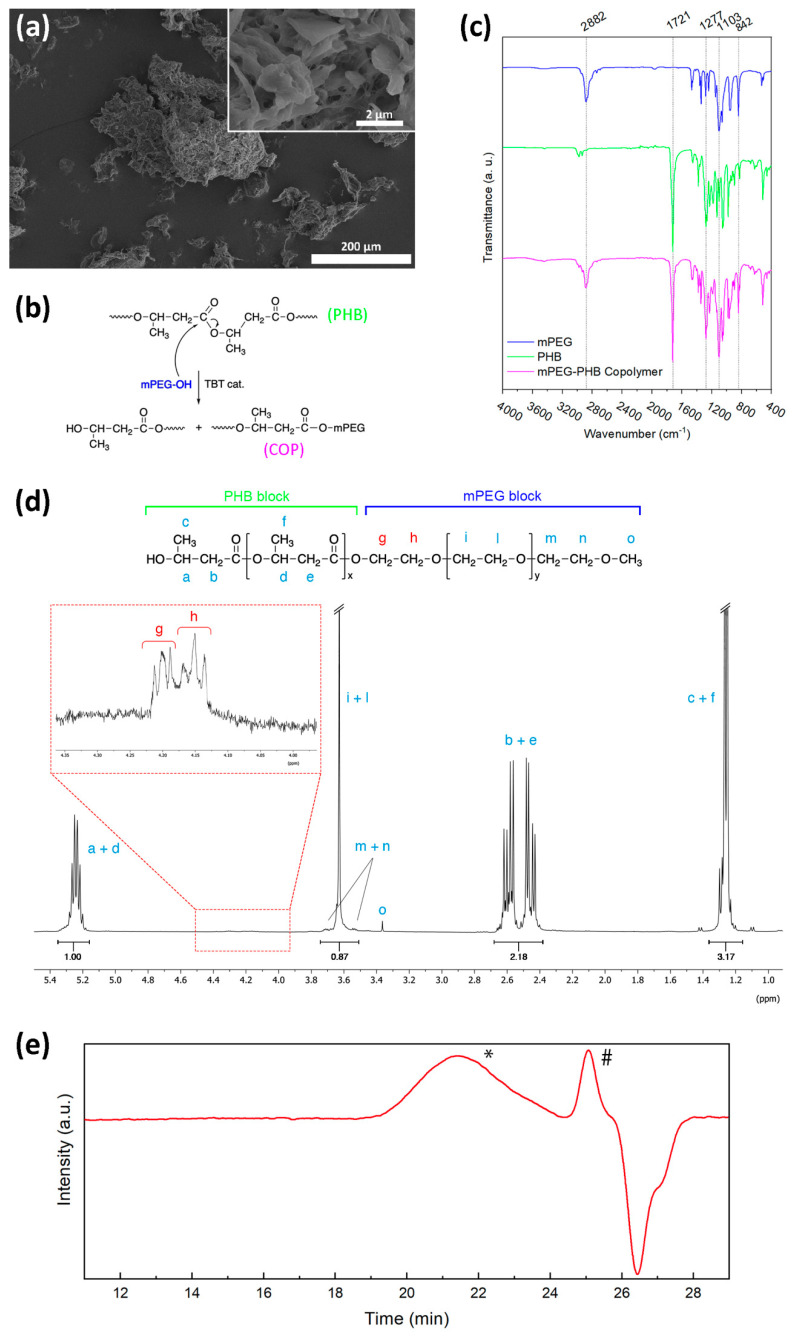
(**a**) FESEM micrographs showing the morphology of the NFC/PEG particles prepared by freeze-drying. (**b**) Scheme of the proposed reaction mechanisms for the copolymer synthesis as previously described by Ravenelle and Marchessault [26]. (**c**) FT-IR spectra of neat PHB, mPEG 1900, and the synthesized PHB–block–mPEG copolymer. (**d**) ^1^H-NMR spectrum of PHB–block–mPEG copolymer and related assignments. The inset highlights the presence of triplets related to the two protons of the newly formed ester bond. (**e**) GPC chromatogram of the synthesized PHB–block–mPEG copolymer. * and # refer to the copolymer and reference peak (toluene), respectively.

The initial part of the study was devoted to the preparation of the dispersible NFC dry fibrils and diblock copolymer. Specifically, the proposed process to produce suitable NFC dry fibrils from NFC suspension forced us to use a polymeric suspending agent to avoid NFC aggregation during water removal. Specifically, we proposed to add a given amount of PEG (formulation detailed in Section 2.2) to the aqueous suspension of NFC, in order to obtain a solid polymer that solidified during the freeze-drying process (experimental details in Section 2.2). This limits the fibril aggregation that typically occurs when the NFC is redispersed in a hydrophobic polymeric material. Hence, the proposed process allowed us to obtain disaggregated NFC fibrils embedded in PEG, as shown in Figure 1a and in the related inset, which can be redispersed in PHB as a filler by an extrusion process.

It is known that PHB/PEG blends have good melt miscibility [28]. On the other hand, PHB/PEG solid blends undergo complex phase separation governed by crystallization conditions, thermal history, and the crystallization rate used for the blend processing stage [28,29]. Therefore, the NFC fibril/PEG requires a compatibilizing agent to be homogeneously redispersed in the PHB. A copolymer composed of blocks of the two polymers was used to compatibilize their interface.

The PHB/PEG copolymer was synthesized according to the reaction scheme reported in Figure 1b, using a single-step solvent-free reaction catalyzed by TBT. In particular, the process was based on trans-esterification, where the only hydroxyl group of mPEG attacks the carboxyl groups of PHB by a mechanism previously described elsewhere [26]. This leads to the breaking of the ester bond of the PHB chains forming the desired copolymer and a lower molecular weight PHB that can react further with another mPEG molecule (Figure 1b).

As shown by the FT-IR curves displayed in Figure 1c, the copolymer spectrum presents the typical signals of both PEG and PHB. Specifically, the band at 2882 cm^−1^ and peaks in the range of 1300–800 cm^−1^ are attributed to the CH_2_ occurring in the PEG structure (stretching, wagging, twisting, and rocking) [30,31]. Moreover, the signal at 1721 cm^−1^, commonly attributed to the carbonyl stretching of the PHB polyester structure [32], is clearly visible. Nevertheless, no signal that clearly confirms the formation of the bond between the two polymers was detected by FT-IR.

In order to confirm the transesterification product, ^1^H-NMR (Figure 1d) was performed. In further detail, in accordance with Ravenelle and Marchessault [26], the detection of the triplet at 4.2 ppm (protons g) representing the two protons from the newly formed ester bond between PHB and mPEG confirmed that the transesterification reaction occurred. Moreover, the ^1^H-NMR investigation allowed us to estimate the block copolymer molar ratio resulting in PHB:mPEG 83:17, calculated by the equations reported in the Supplementary Materials.

GPC analysis revealed that the synthesized copolymer had a number average molecular weight (M_n_) of 2200 g mol^−1^ and a polydispersity index of 2.9 (the GPC curve is displayed in Figure 1e).

**Figure 2:**
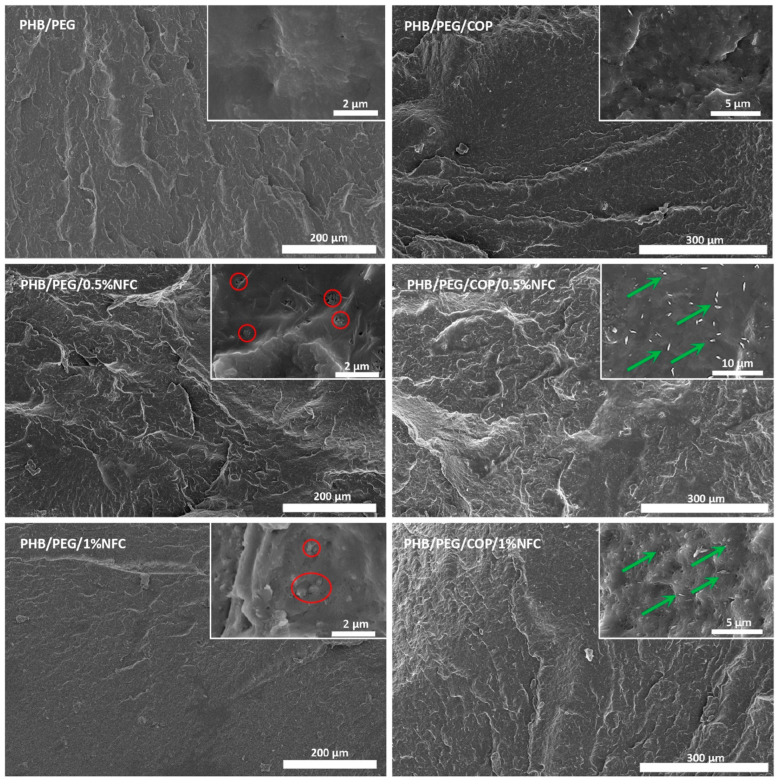
FESEM micrographs (secondary electrons) showing the cross-sections of the two references, PHB/PEG and PHB/PEG/COP, and the related composites with 0.5% and 1% of NFCs, specifically PHB/PEG/0.5%NFC, PHB/PEG/1%NFC, PHB/PEG/COP/0.5%NFC, and PHB/PEG/COP/1%NFC. The insets present higher-magnification images. (Red circles and green arrows highlight NFC aggregates and better exfoliated NFC fibrils, respectively).

After the discussion of the results obtained for the prepared dry NFC fibrils and diblock copolymer, the effect of these two components on PHB’s properties was studied. The cross-sections of the prepared formulations together with neat PHB were investigated by FESEM (Figure 2 and Figure S3) in order to evaluate the NFC distribution in the polymeric matrix. By comparing the NFC-containing formulations, it was possible to observe that the effect of copolymer addition was more evident at the highest NFC content. In particular, several aggregates were detected for NFC composites without the COP. On the contrary, the addition of COP led to better exfoliated NFC fibrils. Moreover, dispersion was improved and only a few aggregates were observed, even when the NFC content increased to 1% (visible in the inset of the micrograph). The combination of these two factors resulted in a wider filler-matrix interfacial area in the composite.

The selected innovative approach for dispersing NFC fibrils starting from their aqueous suspension was effective to prevent particle aggregation and improve filler dispersion.

**Figure 3:**
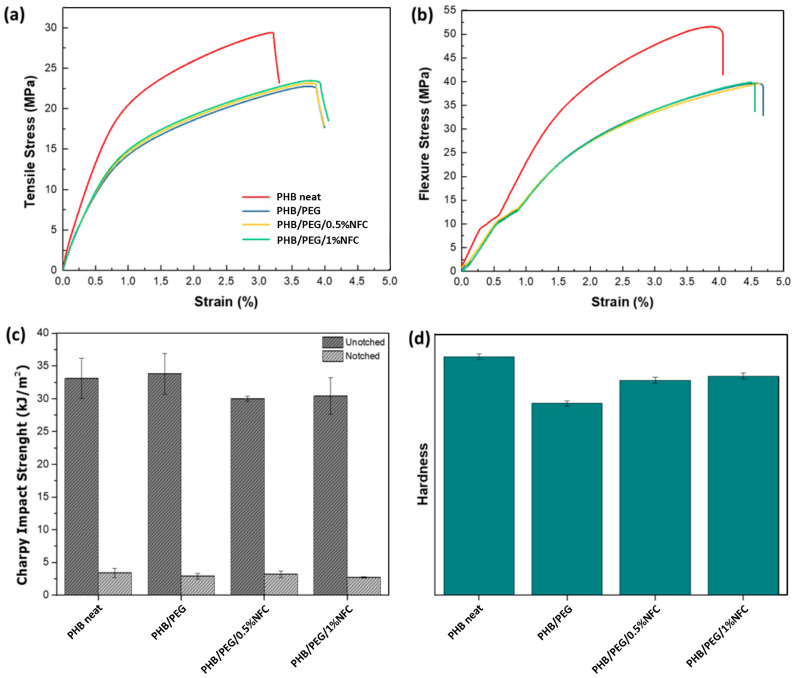
Results of the mechanical tests performed on PHB/PEG/NFC composites: (**a**) stress–strain curves obtained from the tensile analysis; (**b**) stress–strain curves obtained from the flexural test; (**c**) Charpy impact strength and (**d**) Shore D hardness tests.

**Figure 4:**
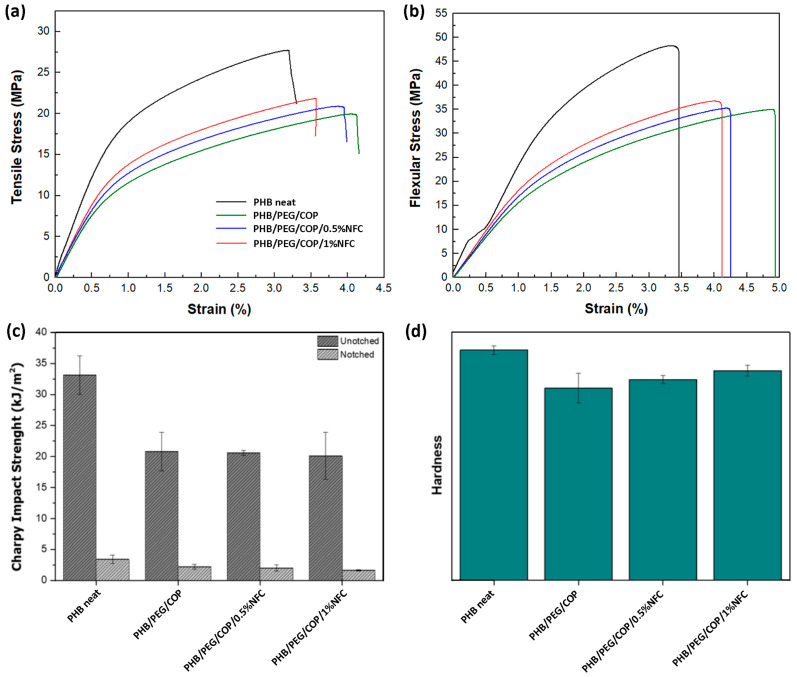
Results of the mechanical tests performed on PHB/PEG/COP/NFC composites: (**a**) stress–strain curves obtained from the tensile analysis; (**b**) stress–strain curves obtained from the flexural test; (**c**) Charpy impact strength and (**d**) Shore D hardness tests.

The mechanical properties of all the formulations were tested by tensile, flexural, and impact analyses, which are presented in Figure 3 and Figure 4 for composites without and with the addition of COP, respectively. Regarding the tensile and flexural behavior of the tested formulations, the well-known plasticizing effect of the PEG can be observed [33]. In particular, Young’s modulus and the elongation at break values of all the PEG-containing formulations, also summarized in Table S1, are characteristic of a less stiff and more ductile behavior if compared to neat PHB. The same trend was also observed for flexural tests. The plasticizing effect of PEG was more evident when the copolymer was added to the formulations, leading to a further decrease in the stiffness and an increase in the elongation at break. In addition, it is worth noting that the fine-tuning of the mechanical properties can be achieved when the NFC is added to the blends. Indeed, by comparing the NFC-containing compositions with and without the compatibilizing COP (Figure 3a,b and Figure 4a,b), a precise adjustment is possible only when COP is present. Moving from PHB/PEG/COP to the 0.5 and 1% NFC-containing composites, Young’s moduli shifted from 1.70 to 1.88 and 1.98 GPa, respectively (a gap of 0.3 GPa, Figure 4 and Table S1). In the absence of COP, the modulus values lay in a smaller range (2.08–2.20 GPa), without a clear trend (Figure 3). By considering the typically observed tensile strength of the bone (35–238 and 1.5–38 MPa for cortical and trabecular bones, respectively) [34], the herein tested PHB/PEG/COP/NFC compositions (19.6–21.7 MPa) were closer to the trabecular bone. On the other hand, their measured stiffness (1.70–1.98 GPa) was one order of magnitude higher, with respect to the range reported for the trabecular bone (0.05–0.1 GPa), and one order of magnitude below the cortical one’s (17–20 GPa). Hence, by modifying the NFC content in the presence of COP in the composite, it was possible to finely tune the stiffness to closely match the desired mechanical properties of the damaged bone.

The same behavior already observed for the tensile tests was also detected for Charpy impact strength test (Figure 3c and Figure 4c). Hardness tests only highlighted the PEG plasticizing effect. A lessening of the hardness was observed when comparing PEG-containing formulations with respect to neat PHB, and no further significant differences were observed among all the formulations (Figure 3d and Figure 4d).

In good agreement with the mechanical properties presented above, the DMA results also show similar trends (Figure S4). Specifically, the flexibility of the prepared materials increases when PEG is present in the compositions. Indeed, storage modulus (E’) values recorded at 50 °C and summarized in Table 2 are approximately halved moving from neat PHB to PEG-containing compositions. Moreover, the formulations without a copolymer present a double T_g_ that is typically ascribable to the poor miscibility of the polymeric components [35] (Table 2). When the copolymer is added to the formulation, a single glass transition is detected, highlighting its compatibilizing effect.

TGA measurements (TGA and DTGA curves reported in Figure S5) were also performed in order to obtain information about the thermal stability of the prepared materials. In particular, it is possible to notice that when PEG is added to PHB, the double-DTG degradation peak of PHB becomes a single peak shifted to a higher temperature. No other significant changes are observed among all the formulations and this effect is even less relevant considering the proposed application in this study.

**Figure 5:**
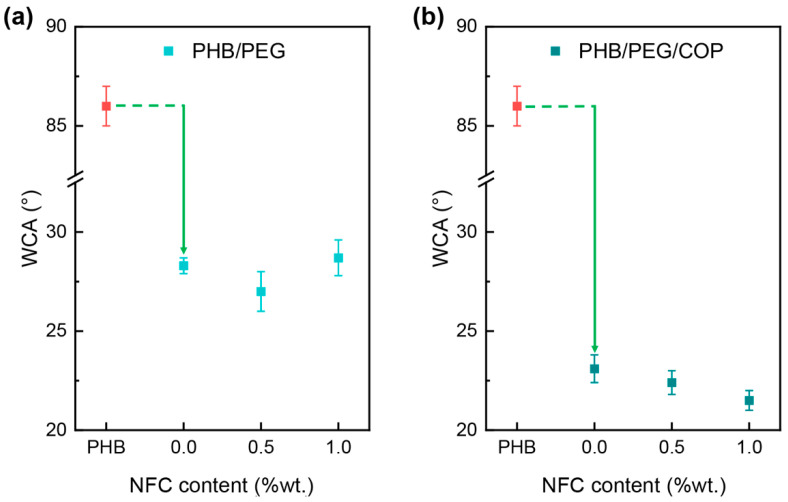
WCA behavior of (**a**) PHB/PEG/NFC and (**b**) PHB/PEG/COP/NFC composites. Green arrows show the WCA decline due to PEG with or without the addition of COP.

The efficacy of the osteointegration process to prevent implant failure is also strictly correlated with the material surface properties, where wettability represents a very crucial factor [36,37]. The effect of NFC and the copolymer’s presence on the wettability of each sample was investigated by static water contact angle (WCA) measurements (Figure 5). The neat PHB sample showed a WCA value of 86°, which is nearly consistent with the values in the literature [38]. The addition of PEG markedly decreases the value to approximately 29°. However, the addition of NFC to PHB/PEG/NFC composites does not produce significant changes in the WCA values. More interestingly, the presence of the copolymer lowers the WCA to 23° and makes the NFC effect more relevant, as the wetting behavior displays a decreasing trend by increasing the amount of NFC. The compatibilizing effect of the copolymer is thus a key aspect for not only tailoring the mechanical properties, but also the wettability behavior of the material’s surface.

## 4. Conclusions

The stress shielding effect is a post-implant surgery drawback that may contribute to implant failure when there is a mechanical mismatch between the bone and implanted material. This well-known problem can be prevented using materials that possess mechanical properties comparable with the treated bone, allowing for a more homogeneous distribution of the loads. In this study, PHB/PEG/NFC composites were prepared by the extrusion process and the compatibilization of different compounds was achieved by the addition of the developed PHB/PEG diblock copolymer. The copolymer was synthesized by a solvent-free synthesis that did not need a complex workup procedure, with a yield close to 100%. Thanks to the copolymer compatibilizing effect, the NFC–PHB interface was improved, resulting in the possibility of obtaining homogeneous composite blends and the precise adjustment of mechanical properties. In particular, it was observed that in the absence of the COP, the elastic modulus values of the blends were similar without showing a clear trend. On the contrary, when the COP is present, by modifying the NFC content in the composite, it was possible to finely tune the stiffness to closely match the desired mechanical properties of the damaged bone since the measured values (1.70–1.98 GPa) were between those of the trabecular and cortical bones (0.05–0.1 and 17–20 GPa, respectively).

Moreover, the homogeneously distributed NFC decreased the typical high water contact angle of PHB (approx. 85–90°) to approx. 22°, allowing us to envision the potential suitability of the modified PHB as supporting material for cell adhesion and proliferation.

Therefore, the materials developed in this study suggest appropriate manufacturers of prosthetic devices to progressively move towards the exploitation of biomaterials with mechanical features that replicate those of bone tissue.

## Figures and Tables

**Table 1:** Formulations of the prepared samples by extrusion.

Sample	PHB (g)	PEG (g)	Copolymer (g)	NFC Compound *	
				NFC (g)	PEG (g)
PHB neat	1000	-	-	-	-
PHB/PEG		40	-	-	-
PHB/PEG/COP		40	50	-	-
PHB/PEG/0.5%NFC		20	-	5	20
PHB/PEG/1%NFC		-	-	10	40
PHB/PEG/COP/0.5%NFC		20	50	5	20
PHB/PEG/COP/1%NFC		-	50	10	40

* NFC compound is composed of PEG/NFC with a wt.%/wt.% concentration of 80/20.

**Table 2:** Storage moduli at 50 °C (E’_T = 50 °C_) and glass transition temperatures (T_g_) of each formulation tested by dynamic mechanical analysis.

Sample	E’_T = 50 °C_ (MPa)	T_g_ (°C)
PHB	2273	11
PHB/PEG	1031	−25/+14 (double T_g_)
PHB/PEG/0.5%NFC	1050	−21/+17 (double T_g_)
PHB/PEG/1%NFC	1054	−22/+13 (double T_g_)
PHB/PEG/COP	1212	9
PHB/PEG/COP/0.5%NFC	1266	12
PHB/PEG/COP/1%NFC	1364	14

## Data Availability

All data are available, on request, from the corresponding authors.

## Supplementary Materials

The following supporting information can be downloaded at: https://www.mdpi.com/article/10.3390/polym15061438/s1, Figure S1: Representative photographs of (a) PHB as received, (b) purified PHB, (c) mPEG 1900, and (d) synthesized PHB–block–mPEG copolymer; Figure S2: Representative photographs of dog-bone specimens of (a) PHB, (b) PHB/PEG, (c) PHB/PEG/0.5%NFC, and (d) PHB/PEG/1%NFC; Figure S3: FESEM micrograph (secondary electrons) of the neat-PHB cross-section. The inclusions detected in the higher-magnification inset can be attributed to cellular residues derived from biological PHB production; Figure S4: (a) Storage modulus (E’) and (b) dissipation factor (Tan δ) with Tg values of PHB/PEG/COP/NFC composites. (c) Storage modulus (E’) and (d) dissipation factor (Tan δ) with Tg values of PHB/PEG/NFC composites; Figure S5: Thermogravimetric analysis curves of (a) TGA and (b) DTGA of PHB, PHB/PEG, and PHB/PEG/NFC composites. Thermogravimetric analysis curves of (c) TGA and (d) DTGA of PHB, copolymer, PHB/PEG, and PHB/PEG/NFC/COP composites. TGA curves were recorded between 25 and 500 °C. DTGA highlights the most significant weight loss between 200 and 450 °C; Table S1: Tensile and flexural mechanical properties of the prepared materials expressed as mean value and related standard deviation.
