# Supplementary material for: Fine Tuning of the Mechanical Properties of Bio-Based PHB/Nanofibrillated Cellulose Biocomposites to Prevent Implant Failure Due to the Bone/Implant Stress Shielding Effect
Source: Polymers (Basel). 2023 Mar 14;15(6):1438. doi: 10.3390/polym15061438 (PMC10051535; doi:10.3390/polym15061438)
Supplement: Supplementary file 1 [file polymers-15-01438-s001.zip › polymers-2222617-supplementary.pdf]

SUPPORTING INFORMATION

Fine Tuning of the Mechanical Properties of Bio-Based PHB/Nanofibrillated Cellulose Biocomposites to Prevent Implant Failure Due to the Bone/Implant Stress Shielding Effect

Martina Ferri [1,2], Emanoele Maria Santos Chiromito [3], Antonio Jose Felix Carvalho [3], Davide Morselli [1,2], Micaela Degli Esposti [1,2,]*, Paola Fabbri [1,2,]*

[1] *Department of Civil, Chemical, Environmental and Materials Engineering, Università di Bologna, Via Terracini 28, 40131 Bologna, Italy*

[2] *National Interuniversity Consortium of Materials Science and Technology (INSTM), Via Giusti 9, 50121 Firenze, Italy*

[3] *Department of Materials Engineering, Engineering School of São Carlos, University of São Paulo, Av. João Dagnone, 1100; 13563-120, São Carlos, SP, Brazil*

* Corresponding Authors

Paola Fabbri
t: +39 051 2090364
p.fabbri@unibo.it

Micaela Degli Esposti
t: +39 051 2090363
micaela.degliesposti@unibo.it

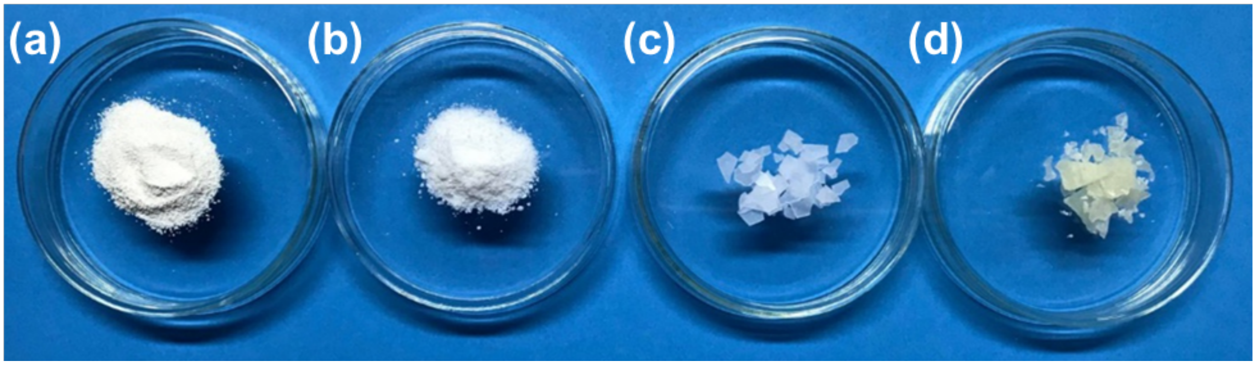

Figure S1. Representative photographs of (a) PHB as received, (b) purified PHB, (c) mPEG 1900 and (d) synthesized PHB-block-mPEG copolymer.

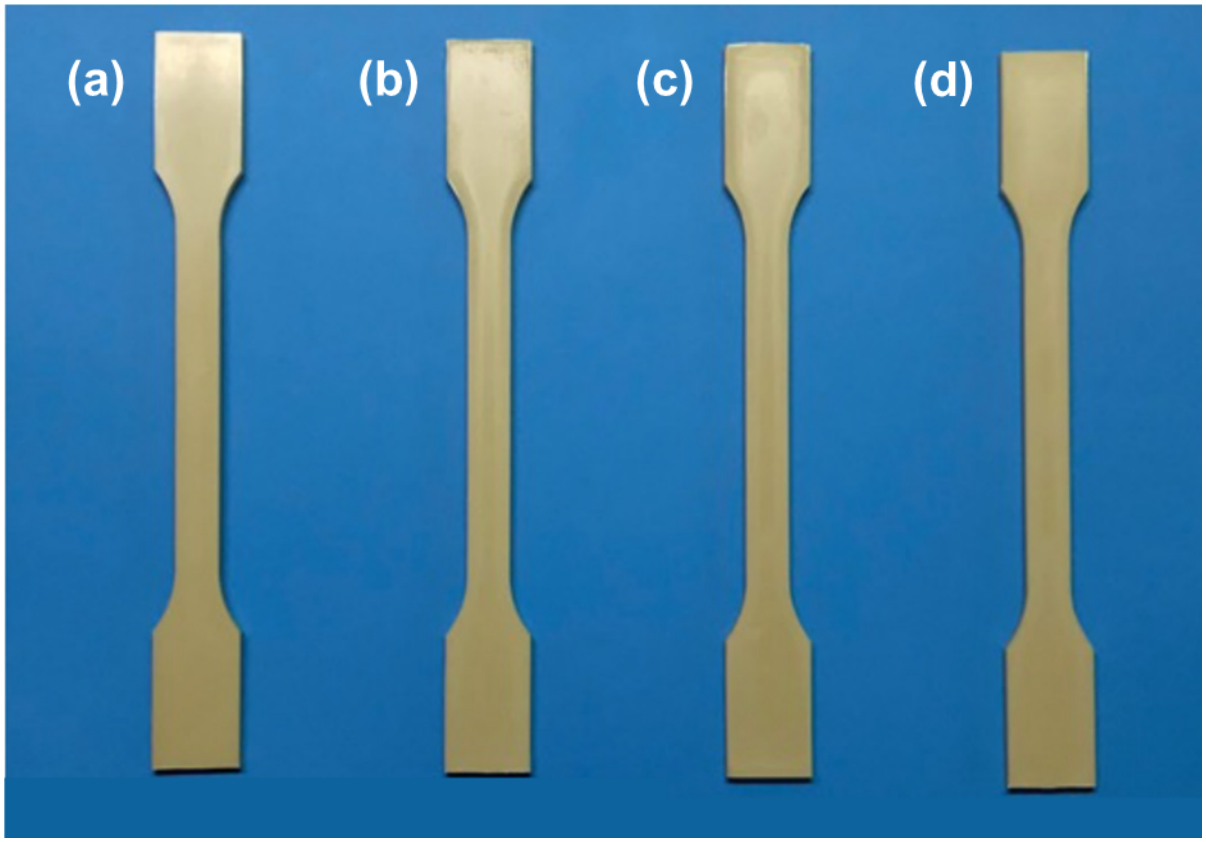

Figure S2. Representative photographs of dog-bone specimens of (a) PHB, (b) PHB/PEG, (c) PHB/PEG/0.5%NFC, and (d) PHB/PEG/1%NFC.

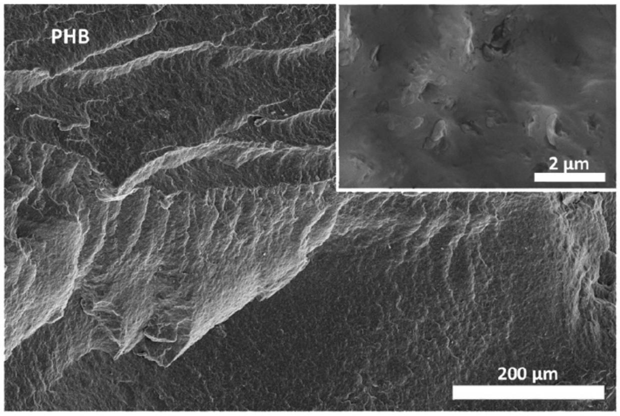

Figure S3. FESEM micrograph (secondary electrons) of the neat PHB cross section. The inclusions detected in the higher magnification inset can be attributed to cellular residues derived from biological PHB production.

Composition of the copolymer blocks

Calculation of the copolymer blocks composition according to Ravenelle and Marchessault [1]. In blue the calculations related to this work and in particular to the ^{1}H-NMR spectrum (Figure 1d of the main paper) are reported.

Hypothesizing that only diblock copolymer chains are present in the synthesized copolymer, the molar fraction of PHB (n_{PHB}) can be calculated as follows:

$$n_{PHB} = \frac{N_{PHB}}{N_{PHB} + N_{mPEG}}$$

where N_x is the average number of repeating units of each segment derived from the normalized ^{1}H-NMR spectrum as:

$$N_{PHB} = \frac{I_{5.3ppm} + I_{2.5ppm} + I_{1.3ppm}}{12}$$

$$N_{PHB} = \frac{2.00 + 4.36 + 6.34}{12} = 1.06$$

$$N_{mPEG} = \frac{I_{3.4\text{-}3.8ppm}}{8}$$

$$N_{mPEG} = \frac{1.74}{8} = 0.22$$

where I_x is the integration value of the peaks and/or regions corresponding to the backbone of each polymer.

By substituting the calculated N_{PHB} and N_{mPEG} values, the molar fraction of PHB (n_{PHB}) results:

$$n_{PHB} = \frac{1.06}{1.06 + 0.22} = 0.83$$

By considering the formula -(HB)$_x$-(EG)$_y$- for the diblock copolymer, it is possible to calculate the corresponding PHB weight fraction:

$$n_{PHB} = \frac{x}{x + y}$$

where y = 1900/44 for mPEG (1900 g mol^{-1} is the mPEG molecular weight and 44 g mol^{-1} is the molecular weight of its monomeric unit). Hence:

$$x = \frac{n_{PHB} * y}{1 - n_{PHB}}$$

$$x = \frac{0.83 * 43.18}{1 - 0.83} = 210.82$$

Since 86 g mol^{-1} is the molecular weight of the monomeric unit of PHB, it is possible to calculate the weight percentage of the PHB block (wt.%$_{PHB}$) as follows:

$$wt.\%_{PHB} = \frac{86 * x}{(86 * x) + 1900} * 100$$

$$wt.\%_{PHB} = \frac{86 * 210.82}{(86 * 210.82) + 1900} * 100 = 90.5$$

Table S1. Tensile and flexural mechanical properties of the prepared materials expressed as mean value and related standard deviation.

	**Tensile properties**			**Flexural properties**		
**Sample**	**Young's Modulus (cord 0.05-0.25 ε%; GPa)**	**Maximum tensile strength (MPa)**	**Elongation at break (ε%)**	**Flexural Modulus (cord 0.05-0.25 ε%; GPa)**	**Maximum flexural strength (MPa)**	**Elongation at maximum flexural strength (%)**
PHB neat	2.62 ± 0.06	27.9 ± 0.1	2.6 ± 0.1	2.36 ± 0.10	51.8 ± 0.8	3.6 ± 0.2
PHB/PEG	2.08 ± 0.04	22.1 ± 0.1	3.6 ± 0.1	1.67 ± 0.10	22.1 ± 0.1	3.6 ± 0.1
PHB/PEG/0.5%NFC	2.20 ± 0.07	23.1 ± 0.1	3.8 ± 0.1	1.71 ± 0.12	23.1 ± 0.1	3.8 ± 0.1
PHB/PEG/1%NFC	2.10 ± 0.06	23.7 ± 0.2	3.8 ± 0.2	1.69 ± 0.26	23.7 ± 0.2	3.8 ± 0.2
PHB/PEG/COP	1.70 ± 0.02	19.6 ± 0.5	4.1 ± 0.2	1.60 ± 0.20	32.7 ± 0.6	4.9 ± 0.1
PHB/PEG/COP/0.5%NFC	1.88 ± 0.04	21.0 ± 0.5	3.9 ± 0.2	1.73 ± 0.10	34.9 ± 0.8	4.3 ± 0.6
PHB/PEG/COP/1%NFC	1.98 ± 0.08	21.7 ± 0.3	3.6 ± 0.4	1.84 ± 0.16	33.7 ± 0.3	4.1 ± 0.2

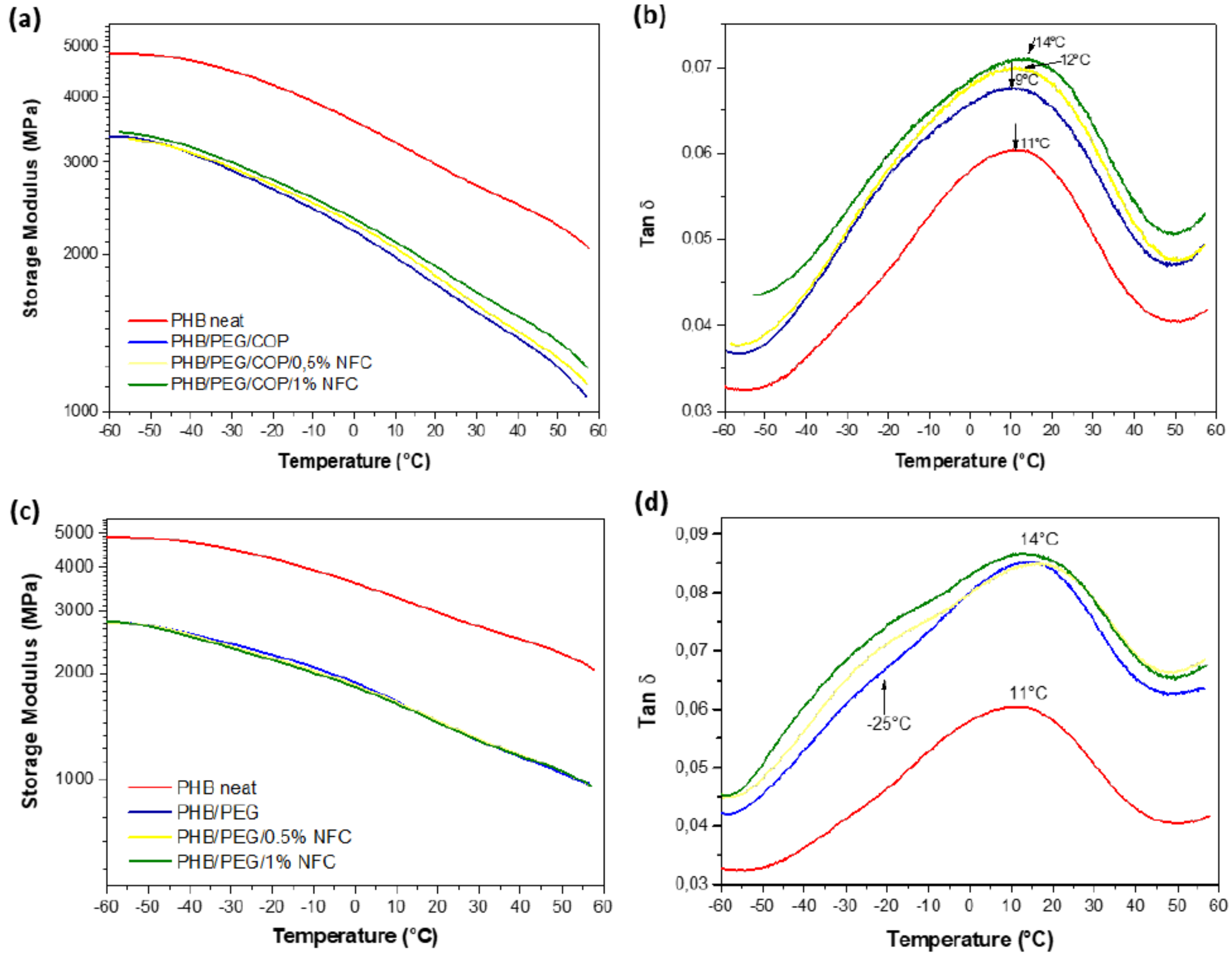

Figure S4. (a) Storage modulus (E′) and (b) dissipation factor (Tan δ) with T_g values of PHB/PEG/COP/NFC composites. (c) Storage modulus (E′) and (d) dissipation factor (Tan δ) with T_g values of. PHB/PEG/NFC composites.

Thermogravimetric analysis experimental details

Thermogravimetric analysis (TGA) was performed on a Perkin Elmer Thermogravimetric Analyzer TGA7. Approx. 10 mg of each sample were heated up to 500°C using a heating rate of 5°C·min^{-1} under nitrogen flow. TGA curves were analyzed by Pyris software.

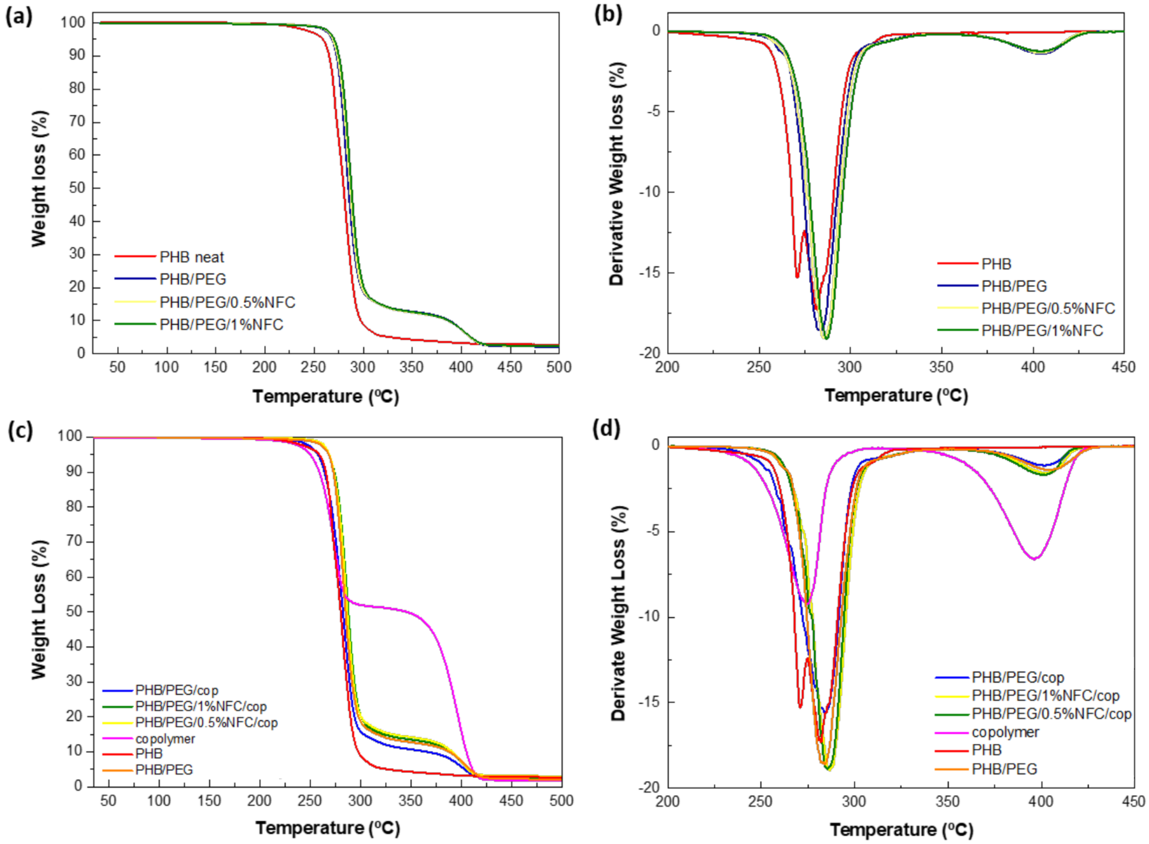

Figure S5. Thermogravimetric analysis curves of (a) TGA and (b) DTGA of PHB, PHB/PEG, and PHB/PEG/NFC composites. Thermogravimetric analysis curves of (c) TGA and (d) DTGA of PHB, copolymer, PHB/PEG, and PHB/PEG/NFC/COP composites. TGA curves were recorded between 25 and 500°C. DTGA highlight the most significant weight loss between 200 and 450°C.

References

1. Ravenelle, F.; Marchessault, R.H. One-Step Synthesis of Amphiphilic Diblock Copolymers from Bacterial Poly([R]-3-Hydroxybutyric Acid). *Biomacromolecules* **2002**, *3*, 1057–1064, doi:10.1021/bm025553b.
